# SERUM LEPTIN LEVENS AND HEPATOCELLULAR CARCINOMA: REVIEW ARTICLE

**DOI:** 10.1590/0102-6720201600040015

**Published:** 2016

**Authors:** Luiza Vitelo ANDRIGHETTO, Aline Kirjner POZIOMYCK

**Affiliations:** 1 Postgraduate Program in Nutrition and Oncology, Institute of Education and Research, Moinhos de Vento Hospital;; 2 Postgraduate Program in Science in Gastroenterology and Hepatology, Federal University of Rio Grande do Sul, Porto Alegre, RS, Brazil

**Keywords:** Hepatocellular carcinoma, Leptin, Adipokine.

## Abstract

**Introduction:**

Hepatocellular carcinoma is one of the most frequent types of malignant tumors in the world. There is growing evidence of the relationship between it development and obesity. The mechanism that links obesity to cancer is still not fully understood; however, it is essential to the understanding the adipose tissue in metabolic changes related to obesity and hepatocellular carcinoma.

**Objective:**

To review the influence of serum leptin levels in patients with hepatocelular carcinoma.

**Method:**

Systematic review of the literature based on the methodology of the Cochrane Institute. The search for articles was in the database: Science Direct, Scielo, Medline, Lilacs e Pubmed. The key words used were hepatocellular carcinoma, leptin, adipokine.

**Results:**

After evaluation of individual studies, were selected seven studies. The results previously studied are still inconsistent and contradictory, and leptin can be effectively involved in the occurrence and development of hepatocellular carcinoma.

**Conclusion:**

Therefore, it is necessary to develop prospective, well-designed and conducted focusing on the role and specific mechanisms of this hormone in patients with hepatocellular carcinoma, so that new correlations can be properly supported.

## RESUMO

**Introdução:**

O carcinoma hepatocelular é um dos tipos mais frequentes de tumores malignos no mundo. Há crescentes evidências da relação entre o seu desenvolvimento e a obesidade. O mecanismo que os relaciona ainda não é completamente entendido. Entretanto é essencial a compreensão do tecido adiposo nas alterações metabólicas relacionadas à obesidade e ao câncer.

**Objetivo:**

Revisar a influência dos níveis séricos de leptina em pacientes com carcinoma hepatocelular.

**Método:**

Trata-se de revisão bibliográfica baseada na metodologia do Instituto Cochrane; a busca de dados foi realizada na base de dados Science Direct, Scielo, Medline, Lilacs e Pubmed, empregando as seguintes descritores: hepatocellular carcinoma, leptin, adipokine.

**Resultado:**

Após avaliação individual dos artigos selecionaram-se sete estudos. Os resultados ainda são inconsistentes e contraditórios, e a leptina pode estar efetivamente envolvida na ocorrência e no desenvolvimento do carcinoma hepatocelular.

**Conclusão:**

Faz-se necessário o desenvolvimento de estudos prospectivos, bem desenhados e conduzidos sobre o papel e mecanismos específicos deste hormônio em pacientes com carcinoma hepatocelular para que novas correlações sejam devidamente comprovadas.

## INTRODUCTION

Hepatocellular carcinoma (HCC) is one of the most frequent types of malignant tumors in the world^8^ ^,^ ^13^. It has the feature to be greatly aggressive, to have very high mortality rate after the onset of symptoms, especially jaundice and/or ascites^7^ ^,^ ^11^. The primary reason for the poor prognosis is the high rate of recurrence and liver failure^6^. When detected late (in the symptomatic phase), the life expectancy is about a month, and the available treatments are limited and ineffective^7^ ^,^ ^11^.

Currently, several methods are used to treat it, depending on the physical conditions and disease staging, for example: surgery, radiofrequency ablation, percutaneous ethanol injection, transcatheter arterial chemoembolization, infusion of arterial chemotherapy, radiation and drug inhibitors class kinases (Sorafenib). Liver transplantation is the last choice therapy, but this strategy is limited due to the shortage of donors. All current treatment modalities can cause hepatic and systemic damage and, in many cases, treatment becomes more difficult due to the high prevalence of these cirrhotic patients^14^. Can be highlighted as important risk factors for the development of HCC: infections caused by hepatitis B or hepatitis C and excessive alcohol consumption^4^.

There is growing evidence of the relationship between the development of HCC and obesity^17^. Epidemiological studies have linked overweight and obesity with increased risk of HCC compared to the general population, and higher mortality^3^ ^,^ ^5^. The mechanism that links obesity to HCC is still not fully understood; however, it is essential to the understanding of adipose tissue in metabolic changes related to obesity and HCC^20^. 

Recently, adipose tissue has been considered an endocrine organ to produce a variety of biologically active adipocytokines such as leptin, adiponectin, and resistin. Therefore, the unregulated expression of these adipocytokines may be involved in the association of obesity with the development of HCC^12^ ^,^ ^15^.

Due to inconclusive studies on the influence of adipose tissue and its adipocytokines in association with the HCC, the present study aims to review the influence of serum leptin in patients with hepatocellular carcinoma.

## METHODS

This is a systematic review of the literature based on the methodology of the Cochrane Institute^10^. To carry out the study, the following steps were taken: 1) formulation of the research question; 2) location and selection of studies; 3) critical appraisal of the studies; 4) data collection; 5) data analysis; 6) interpretation of data; 7) improvement and update of the review.

Were systematically searched Science Direct, Scielo, Medline, Lilacs and Pubmed selecting the publications available between 1996 and 2016, in Portuguese, English and Spanish using the following headings (used individually or in combination): hepatocellular carcinoma, leptin, adipokine, hepatocellular carcinoma, leptin, adipocitoquina and their abbreviations. The search for articles included in this study was conducted in the period January to May 2016

Clinical papers were included if they fulfilled the following criteria: contemplated the serum leptin levels and hepatocellular carcinoma; published in Portuguese, English or Spanish; review, observational. No defined methodology articles were excluded, as well as studies conducted in animals and in vitro.

They were evaluated for the research designs, types of analysis and sample size. The outcome was the relationship of serum leptin and HCC. 

**FIGURE 1:**
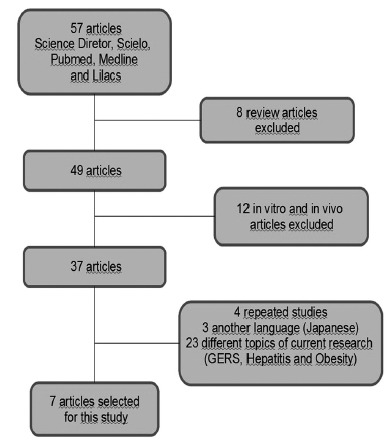
Flowchart of studies demonstrating the selection process

The search results were screened independently by two qualified nutritionists using titles of the articles and their summaries. After identification of relevant studies, a complete publication was acquired and evaluated independently by two authors to determine eligibility for final inclusion in this study, based on pre-selected selection criteria (Figure 1). 

## RESULTS

In electronic search, 57 publications were obtained, which, after reading the abstracts and exclusion of articles that contemplated some exclusion criteria, were included seven articles, described in Table 1.

**TABLE 1:** Characteristics of included studies

Author /Year	Study design	Analysis	Number (Cases / controls)	Main results
Wang/ 2003	Case control	Serum leptin level in HCC	31 Cirrhotic + HCC 26 Cirrhotic 25 Controls	Increased serum leptin in cirrhotic patients with or without HCC
Wang/2006	Cohort	Leptin expression in HCC	68 HCC	High expression of leptin associated with intra- tumor MVD increase and increased survival
Wang/2006	Cohort	OBR expression in HCC	66 HCC	High expression of the OBR was positively correlated with MVD and better overall survival, and inversely correlated with vascular invasion
Ataseven/ 2006	Case control	Serum leptin level in HCC	23 Cirrhotic 25 HCC 25 Control	Lower serum leptin levels in patients with cirrhosis and HCC co- infected with hepatitis B and D compared to the control
Miyaha/ 2011	Prospective Cohort	Serum leptin level in HCC	30 HCC treated with Sorafenib	Elevated levels of serum leptin were associated with little effect on treatment with Sorafenib in patients with HCC
Watanabe/ 2011	Prospective cohort	Serum leptin level in HCC	85 HCC in primary care	Increased serum levels of leptin was a risk factor for recurrent phase I/II of HCC after curative treatment
Sadik/2012	Retrospective cohort	Serum leptin level in HCC	19 HCC not cirrhotic 50 HCC cirrhotic 36 Cirrhotic 21 Control	The higher serum leptin in HCC patients in both groups, but there was no difference between the group with cirrhosis compared with normal controls

HCC=hepatocellular carcinoma; OBR=leptin receptor; MVD=intratumoral microvascular density

## DISCUSSION

Since it was first described in 1994, leptin is the most studied adipokines as well as its association with obesity gene^26^. Leptin acts on the hypothalamus and is responsible for the control of food intake. Its action in the central nervous system promotes the reduction of food intake and increased energy expenditure, regulates hematopoiesis, lipid metabolism and glucose and regulate the immune and inflammatory response^9^. The increase in leptin production that occurs during inflammation and infection suggests that leptin part of the cytokine cascade. Recently, the study focus has been on its role in liver fibrosis in animal models^1^.

A study conducted on mice in 2001, by Yang et al^25^ explored for the first time if obesity could increase the risk of HCC, concluding that the ob/ob mice (obese) developed hyperplasia in the liver stage more early non-alcoholic fatty liver disease evolving to HCC. This association speculated the possibility of fatty liver in obese patients is a premalignant condition. Wang et al^23^ investigated the involvement of leptin in the etiology of HCC in cirrhotic patients. This study noted that the increase in serum leptin was significantly correlated with cirrhosis (p<0.005), in patients with cirrhosis had higher serum levels of leptin than the controls, but not related to the development of HCC. However, the study by Sadik et al^19^, serum leptin levels were normal in groups of patients with cirrhosis without HCC and controls, although in patients with HCC, serum levels were as high. Therefore, they concluded that whether cirrhotic serum leptin levels had to be high in patients with HCC, is suggested larger prospective studies on the relationship between leptin and the HCC.

Ataseven et al^2^ in a study of 23 patients with cirrhosis and 22 with HCC and all co-infected with hepatitis B and D compared to 25 controls (healthy subjects), they found lower serum leptin levels in patients with cirrhosis and HCC compared to healthy subjects (p<0.05), and high levels of ghrelin in both groups. However, when comparing the cirrhosis groups and HCC, there was no significant difference for both hormones, which indicates that this change can be for metabolic disorders due to cirrhosis and not the HCC, since our patients who had HCC were classified as ChildPug - C (n=11, 50%). It is known that insulin resistance is present in cirrhosis caused by hepatitis C virus - uncommon in other viral hepatitis - and this resistance would consequently have to explain the high levels of leptin. It is speculated that precisely because it is a rare situation in viral hepatitis B and D, this result may be a consequence of malnutrition and adipocytes reduction in patients infected with hepatitis B or D cirrhotic instead of insulin resistance installed by infection.

A study carried out in Okayama^16^ evaluated the influence of serum leptin levels as a biomarker of the effect of treatment with Sorafenib in patients with HCC. The authors found that high levels of serum leptin at start of treatment were correlated with lesser effect of Sorafenib. Progression-free survival was significantly lower in patients with high levels of leptin, with a hazard ratio of 4.14. In another analysis of prospective cohort by Waranabe et al^24^ demonstrated that the increase in leptin levels was an independent risk factor for recurrence (p=0.0035 phase I/II after curative treatment by surgical resection or ablation radiofrequency. This finding indicates that the increase in serum leptin concentration can connect obesity with carcinogenesis of liver cancer, is a good biomarker for screening high risk of recurrence groups.

Increased leptin expression is also associated with increased intratumoral microvessel density (MVD). Consequently, there is the possibility that leptin by neovascularization has a stimulatory role in the development of HCC^18^. Furthermore, studies developed by Wang et al^21,22^ evaluated the expression of leptin and its receptor (BR) for HCC sample by immunostaining also correlated with Ki-67 expression profile (tumor proliferation marker), MVD and overall survival, provided by clinical evidence on prognostic roles of leptin and OBR. First, the OBR expression was inversely correlated with vascular invasion of HCC.

In addition, high expression of leptin was associated with improved survival in HCC patients postoperatively treated with medroxyprogesterone acetate (orexigenic agents - associated with increased appetite), a synthetic variant of human progesterone. Thus, it can be suggested that both high levels of serum leptin, as the high expression of its receptor in HCC tissues could be better overall survival prognosis .

## CONCLUSION

The results previously studied are still inconsistent and conflicting and leptin can effectively be involved in the occurrence and development of HCC. Therefore, it is necessary to develop prospective, well-designed and conducted on the role and specific mechanisms of this hormone in patients with HCC, so that new correlations can be properly supported.
